# Utilising artificial intelligence for cultivating decorative plants

**DOI:** 10.1186/s40529-024-00445-9

**Published:** 2024-12-19

**Authors:** Nurdana Salybekova, Gani Issayev, Aikerim Serzhanova, Valery Mikhailov

**Affiliations:** 1 Department of Biology, Khoja Akhmet Yassawi International Kazakh-Turkish University, Turkistan, Kazakhstan; 2 Department of System Analysis and Information Technologies, Kazan Privolzhsky Federal University, Kazan, Russian Federation

**Keywords:** ANFIS, Artificial neural networks, Decision-making, Integrated pest management, Risk assessment, Tulips

## Abstract

**Background:**

The research aims to assess the effectiveness of artificial intelligence models in predicting the risk level in tulip greenhouses using different varieties. The study was conducted in 2022 in the Almaty region, Panfilov village.

**Results:**

Two groups of 10 greenhouses each (area 200 m2) were compared: the control group used standard monitoring methods, while the experimental group employed AI-based monitoring. We applied ANOVA, regression analysis, Bootstrap, and correlation analysis to evaluate the impact of factors on the risk level. The results demonstrate a statistically significant reduction in the risk level in the experimental group, where artificial intelligence models were employed, especially the recurrent neural network “Expert-Pro.” A comparison of different tulip varieties revealed differences in their susceptibility to risks. The results provide an opportunity for more effective risk management in greenhouse cultivation.

**Conclusions:**

The high accuracy and sensitivity exhibited by the “Expert-Pro” model underscore its potential to enhance the productivity and resilience of crops. The research findings justify the theoretical significance of applying artificial intelligence in agriculture and its practical applicability for improving risk management efficiency in greenhouse cultivation conditions.

## Background

Innovations in artificial intelligence (AI) have rapidly expanded its applications across various domains, with notable advancements in ornamental horticulture—where precision, sustainability, and responsiveness are essential (Tay et al. 2021). Traditional plant care methods often rely on fixed schedules for irrigation, fertilization, and pest management, yet they lack the real-time adaptability needed to respond to nuanced environmental changes. AI addresses these limitations, introducing a new level of precision by utilizing data-driven insights and dynamic management strategies in greenhouse settings (Farias et al. 2022).

In greenhouse cultivation of decorative plants, AI-powered tools such as artificial neural networks (ANNs) enable automated monitoring and predictive control systems that assess plant conditions in real time. These intricate algorithms mimic human cognition, analyzing data from multiple sensors to detect early signs of stress, disease, and pest presence, which are otherwise challenging to identify promptly (Singh et al. 2022). AI enhances pest control, a particularly critical aspect, especially for delicate varieties like tulips, which are highly susceptible to infestations that can degrade their aesthetic value (Apushev et al. 2023).

AI’s potential to support plant health management extends beyond pest control. By optimizing irrigation and nutrient supply, AI systems help reduce water and fertilizer usage, supporting sustainability and mitigating environmental impacts. Research increasingly demonstrates that AI can streamline these processes, making them more efficient and eco-friendly, thus aligning with the goals of sustainable agriculture (Safonov et al. 2019).

Despite the promise AI holds, several challenges remain in integrating these technologies into horticultural practices. For example, adapting AI models to manage the environmental variables in greenhouses and predict pest behavior across different plant species requires more empirical testing and adjustment (Lim et al. 2023). Current studies also show the importance of fine-tuning AI models to account for specific plant varieties’ responses to environmental factors, such as temperature and humidity (Mahmud et al. 2023). Addressing these factors ensures AI models are effective across diverse horticultural contexts.

The importance of AI research in ornamental horticulture can be categorized into three primary areas. First, AI-driven optimization of plant care directly enhances production efficiency, reducing the cost of resources and improving yield while minimizing the ecological footprint of decorative plant cultivation (Tay et al. 2021; Singh et al. 2022). Second, AI-based monitoring systems allow for early detection of disease and pest activity, which is essential in mitigating crop losses and maintaining plant health. By proactively addressing these issues, AI contributes to the resilience of horticultural systems (Farias et al. 2022). Finally, AI’s role in managing the reproduction and diversity of decorative plant varieties supports biodiversity and offers unique aesthetic opportunities for landscaping and gardening (Farias et al. 2022; Oberti and Schmilovitch 2021).

In summary, the adoption of AI in the cultivation of decorative plants not only advances agricultural practices but also enhances environmental protection, pest management, and aesthetic diversity. This holistic approach to horticulture exemplifies AI’s transformative potential, laying a foundation for future research and development in sustainable and intelligent agriculture.

### Literature review

One of the key applications of AI in agriculture is the early detection of pests in greenhouse settings, a critical factor in enhancing crop resilience and reducing yield losses. AI-based systems enhance monitoring by automating pest detection processes and identifying anomalies in plant behavior through sensor analysis. These systems allow for immediate responses to changes in pest activity, making them valuable not only in greenhouses but also in open fields, thereby broadening the scope of AI applications in agriculture (Lim et al. 2023; Safonov 2023).

A significant aspect of AI’s application in horticulture lies in its reliance on artificial neural networks (ANNs) for data processing and pattern recognition. ANNs have shown high efficacy in identifying subtle patterns and trends that may not be immediately visible to the human eye. When applied to pest control, ANNs help recognize changes in plant responses that indicate physiological disturbances caused by pests, supporting early and targeted intervention measures (Hammam and Adly 2022).

The integration of AI technologies in agriculture not only improves the efficiency of monitoring but also reduces the dependency on chemical pesticides. This shift is essential for promoting sustainable agriculture, as it minimizes environmental impact while ensuring high yields. The application of AI for pest detection represents a step forward in modern agricultural practices, where precision and timely intervention are vital for maintaining plant health and quality (Li et al. 2022; Singh et al. 2022). This perspective aligns with recent studies on reducing pesticide use and improving resource efficiency through AI-driven precision agriculture (Mahmud et al. 2023).

To monitor plant health, AI relies on diverse technologies, including advanced sensors, cameras, and automated data processing systems. Sensors such as temperature, humidity, and light sensors are crucial for real-time environmental monitoring in greenhouses. This data, transmitted to a central AI-powered system, allows for precise control of the cultivation environment. Video cameras capture changes in plant growth and appearance, while computer vision and image analysis systems detect anomalies, such as color, shape, or texture changes indicative of pest impact (Rustia et al. 2022).

Data collected by these devices is then processed using ANNs trained to recognize patterns in plant health. These networks provide early warnings by identifying deviations associated with pest activity, enabling timely and preventive measures. Such automated responses may include adjusting irrigation, applying fertilizers, or even biological control interventions, such as the introduction of natural predators to target specific pests (Coulibaly et al. 2022; Cuong et al. 2022).

In recent years, other sensor types have expanded the toolkit for greenhouse monitoring. For instance, spectrometers help measure light absorption, revealing photosynthetic activity levels that can indicate plant stress. Acoustic sensors monitor sound emissions, which may signal pest presence, while electrophysiological sensors track electrical changes in plants, which could suggest pest-induced stress (Branding et al. 2023). Additionally, drones equipped with thermal and multispectral imaging collect aerial data to detect growth anomalies across extensive crop areas (Domingues et al. 2022).

Moreover, external factors like weather conditions gathered from meteorological stations are vital for predicting risks such as frost or heatwaves. These combined monitoring tools provide detailed data on the cultivation environment and plant health status. Analyzing this data with AI algorithms allows for early issue detection and ensures efficient crop management, supporting agricultural sustainability (Toscano-Miranda et al. 2022).

Among the most popular ornamental plants, tulips are notable for their wide variety and aesthetic appeal, but they are also susceptible to a range of pests. Over 3000 tulip varieties are classified based on shape, height, and color, each requiring specific soil conditions. Tulip cultivation in greenhouses is particularly vulnerable to infestations from insects and mites, including thrips (Thysanoptera), caterpillars (Lepidoptera), and flower thrips (*Frankliniella *spp.), each causing unique types of damage that affect plant quality (Cheng et al. 2022; Jarecka-Boncela et al. 2023).

To combat these pests, greenhouses use biological methods, chemical agents, and climate management. Biological control measures, such as introducing predators, complement traditional pest control methods, yet the risks of plant damage from chemicals and pest resistance to insecticides remain challenges. These challenges underscore the importance of exploring AI’s role in early pest detection to mitigate infestations before they proliferate on a large scale (Silva 2023; Oberemok et al. 2023).

The combined use of these modern AI tools and diverse sensor technologies in greenhouse management represents a substantial innovation in pest control and plant health monitoring. However, despite advancements, additional empirical testing is essential for optimizing AI model performance, addressing specific crop characteristics, and improving adaptation to diverse environmental variables.

### Research tasks

The primary objective of this study is to develop and evaluate an AI-driven system for predicting pest risk levels in tulip greenhouses by leveraging artificial neural networks (ANNs) and an adaptive neuro-fuzzy inference system (ANFIS). Given that pest infestations present a significant threat to tulip cultivation, particularly in greenhouse environments, timely detection at the early stages of plant development is essential for preventing substantial agronomic and economic losses. Traditional monitoring approaches often lack the precision and responsiveness required to manage the complexities of pest behavior, which can be influenced by various environmental factors. This study aims to address these limitations by implementing advanced AI technologies, specifically ANNs, and ANFIS, to optimize monitoring and improve pest control strategies in tulip greenhouses.

### Relevance of the study

This research addresses a critical need within horticulture and agriculture for effective, scalable, and environmentally friendly pest management solutions. The complexities associated with pest development—such as varying climate conditions, plant susceptibility, and pest resistance—demand sophisticated monitoring techniques. By implementing AI-based systems, this study not only enhances the effectiveness of pest management in ornamental plant cultivation but also contributes to sustainable practices by reducing dependency on chemical pesticides and optimizing resource use. Furthermore, as global demand for ornamental plants like tulips continues to grow, efficient pest management is essential for meeting industry standards and ensuring high-quality crop yields (Safonov et al. 2019; Tay et al. 2021). Thus, this research provides both practical and theoretical contributions, offering a replicable AI-based approach for the broader horticultural industry.

### Research objectives

To achieve the outlined goals, the study is structured around the following specific objectives: (a) To evaluate the comparative effectiveness of pest detection using conventional methods versus AI-driven approaches (ANN and ANFIS) within tulip greenhouses, quantifying improvements in detection speed, accuracy, and response time; (b) To analyze the strengths and limitations of AI-based monitoring methods, with a focus on resource efficiency, environmental impact, and long-term applicability for greenhouse management; (c) To examine the influence of key environmental factors (e.g., temperature, humidity) on pest risk levels and assess how the ANN and ANFIS models can adapt to these variables in real-time; (d) To develop recommendations for integrating AI systems in ornamental horticulture, providing practical guidelines for implementing AI-based pest management solutions at a commercial scale.

## Methods and materials

### Research subject

This study was conducted in 2022 in the Panfilov settlement, Almaty Region. The experiment involved 20 greenhouses from a major farm, each measuring 200 m². A randomized selection process was used to assign 10 greenhouses to the control group, where traditional pest monitoring techniques were employed, and the remaining 10 to the experimental group, which utilized artificial intelligence (AI) tools, specifically artificial neural networks (ANNs) and an adaptive neuro-fuzzy inference system (ANFIS), for pest monitoring. This randomized allocation was intended to eliminate potential biases and ensure balanced environmental conditions across both groups.

The research focused on tulip varieties commonly cultivated for decorative purposes: Albatros, Delta Storm, Fun for Two, Strong Power, and Dynasty. These varieties were selected based on their popularity in commercial horticulture and known susceptibility to common greenhouse pests, making them ideal for testing AI-based monitoring systems. Bulbs were pre-processed (cleaned, sorted by size) and stored under controlled temperature conditions before planting on December 30, ensuring uniformity in initial growth conditions.

### Study design

Standard monitoring methods in the control group included:

Conducted by a trained team, Regular Visual Inspections identify physical signs of pest damage, such as changes in leaf color, deformation, or visible pests. To reduce observational bias, the same team performed all inspections, which increased in frequency during peak pest activity periods, particularly at the beginning of the growing season and during the flowering phase.

Sticky traps were used to capture insects at the plant level, with inspections ranging from once to twice weekly. Pheromone traps, set at the same frequency, were placed strategically around plants to attract and capture pests, providing additional data on population dynamics.

Temperature and humidity sensors were installed in each greenhouse to track environmental conditions conducive to pest reproduction. Data were recorded hourly and analyzed for seasonal patterns that could influence pest behavior.

### Research methods

The experimental group employed an AI-driven monitoring system, using ANNs and ANFIS to assess risk levels. This system processed four main explanatory variables: internal temperature, internal humidity, phytosanitary risk, and human intervention frequency. The goal was to create a real-time risk profile to guide pest management responses effectively.

The system used a network of strategically placed sensors and cameras to monitor the environmental and physiological conditions of the tulips:

Temperature sensors were evenly distributed throughout the greenhouse, positioned at heights between 0.5 and 1 m above ground to measure plant-level conditions accurately. Humidity sensors were similarly positioned across different zones, accounting for air layer variations and proximity to the soil.

Optical cameras monitored tulip health during daylight hours, while infrared cameras enabled nighttime surveillance. Cameras were installed at regular intervals to ensure complete coverage, positioned approximately 2 m apart to capture detailed images of plant health and pest presence.

Sensor and camera data were continuously transmitted to a central AI-based monitoring system. Using pre-trained ANN models, this system analyzed incoming data to detect risk factors and potential pest activity in real time. The ANFIS model further optimized predictions by adjusting parameters based on observed environmental variations and pest population changes.

### Statistical analysis

To analyze the impact of AI on pest control efficiency, the following statistical methods were applied: (a) Independent Samples t-Test: Used to compare mean risk levels between control and experimental groups, assessing the statistical significance of differences observed between the two approaches. (b) Analysis of Variance (ANOVA): Employed to evaluate variations in risk levels across different tulip varieties within each group. Post-hoc tests identified specific pairs of varieties with statistically significant differences. (c) Regression Analysis: Performed to understand the influence of temperature, humidity, phytosanitary risk, and human intervention on risk levels, thus contributing to a predictive model for risk levels based on environmental factors. (d) Bootstrap Methods: These methods were applied to estimate confidence intervals for key outcomes, accommodating sample variability and ensuring robust result interpretation. (e) Correlation Analysis: Used to assess the relationships between explanatory variables and the risk level, identifying which factors most strongly correlated with pest presence.

### Additional considerations

To improve the interpretability of the data, standard error values were included for key indicators in all tables, as recommended. Moreover, clear criteria for selecting greenhouse groups and conditions for sensor calibration were established to maintain measurement consistency across greenhouses. By implementing these methods, this study provides a rigorous evaluation of AI and ANFIS models in practical, real-world conditions.

### Results

The generalized analysis of risk levels in the groups demonstrates a substantial reduction in the mean risk level in the experimental group compared to the control. Statistical differences were confirmed using the t-test (*p* < 0.05, Table 1).

**Table 1:** Overall risk level in groups^*^

Group	Average risk level	Standard deviation	Standard error
Control	25.3	3.2	0.7
Experimental	20.1	2.8	0.6

Standard errors have been added to provide greater precision in comparing the control and experimental groups

^*^ - in Tables 1, 2, 3, 4 and 5, units are measured in percentages

A detailed analysis of tulip varieties revealed statistically significant differences in the risk level between the control and experimental groups for the Albatros and Delta Storm varieties (*p* < 0.05, Table 2). For the remaining varieties, the differences did not reach the level of statistical significance.

**Table 2:** Analysis of risk level by tulip varieties

Tulip variety	Control	Experimental	Difference (*p*-value)
Albatros	22.5	18.2	0.034
Delta storm	24.8	20.5	0.042
Fun for two	21.3	19.8	0.312
Strong power	23.1	21.7	0.189
Dynasty	20.7	19.3	0.275

In the experimental group, a significant reduction in infestations of thrips, leafrollers, and mites is observed compared to the control group (*p* < 0.05, Table 3).

**Table 3:** Infestation by pests in groups

Group	Thrips (%)	Leafhoppers (%)	Mites (%)
Control	15.2	7.6	3.1
Experimental	11.5	5.2	2.8

The analysis of differences in the risk level at different stages of the vegetative cycle revealed a statistically significant reduction in risk in the experimental group during the growth, development, and flowering phases (*p* < 0.05, Table 4).

**Table 4:** Analysis of the risk level at different stages of the vegetative cycle

Cycle Stage	Control	Experimental	Difference (*p*-value)	Explanation
Growth and development	24.0	20.5	0.018	Early growth phase where plants are most vulnerable to pest establishment and initial infestations.
Budding	22.2	19.8	0.092	During budding, plant structures begin to develop, and pest presence may affect bud formation, slightly decreasing overall risk.
Flowering	20.5	18.0	0.034	In this stage, pests can directly affect flower quality; lower risk indicates successful early pest management.
Fruiting and ripening	18.7	16.2	0.062	Final stage of development, where pest impact on yield quality and quantity is minimized through continuous monitoring and intervention.

Detailed descriptions for each phase clarify how pest risk levels shift based on plant vulnerability during the growth cycle

The investigation of the dynamics of the risk level across different periods revealed statistically significant differences in the risk level between the groups during period 1 (*p* < 0.05, Table 5).

**Table 5:** Dynamics of the risk level over time

Time period^*^	Control	Experimental	Difference (*p*-value)
Period1	21.3	18.7	0.041
Period2	23.1	20.8	0.075

Period 2 - later stages of the vegetative cycle, the phase of intensive growth and bulb formation

^*^period 1 - early stages of the tulip vegetative cycle, the budding phase, and the beginning of flowering

The correlation coefficients indicate a significant influence of temperature and humidity on the risk level (*p* < 0.05, Table 6). Other factors, such as phytosanitary risk and human intervention, do not have a statistically significant impact.

**Table 6:** Correlation between factors and risk level

Factor	Coefficient of correlation	*p*-value
Temperature	0.65	0.002
Humidity	0.48	0.032
Phytosanitary risk	− 0.12	0.589
Human intervention	0.27	0.125

Results of comparing the effectiveness of three artificial intelligence models in predicting the risk level indicate that Model 3 (“Expert-Pro”) outperforms other models in all considered metrics (*p* < 0.05, Table 7).

**Table 7:** Comparison of artificial intelligence models’ effectiveness in predicting risk levels in tulip greenhouses

Indicator	Group	Model 1	Model 2	Model 3
(Accuracy)	Control	0.75	0.78	0.81
	Experimental	0.82	0.87	0.89
(Sensitivity)	Control	0.68	0.72	0.75
	Experimental	0.79	0.84	0.88
(Specificity)	Control	0.82	0.86	0.89
	Experimental	0.89	0.91	0.93
Positive predictive value (PPV)	Control	0.77	0.81	0.84
	Experimental	0.85	0.88	0.91
Negative predictive value (NPV)	Control	0.83	0.87	0.90
	Experimental	0.90	0.92	0.94
Area under the ROC curve (AUC-ROC)	Control	0.88	0.91	0.94
	Experimental	0.92	0.94	0.96
F1-score	Control	0.72	0.76	0.79
	Experimental	0.81	0.85	0.88

Model 1 - Neural Network “Predictor,” Model 2 - Convolutional Neural Network “Precision+,” Model 3 - Recurrent Neural Network “Expert-Pro”

The conducted ANOVA revealed statistically significant differences in the risk levels among tulip varieties in each group (*p* < 0.05). Post-hoc tests confirmed that differences between specific pairs of varieties, such as Albatros and Delta Storm (*p* = 0.034) or Delta Storm and Strong Power (*p* = 0.042), are statistically significant. Regression analysis showed that internal temperature has a positive correlation with the risk level (correlation coefficient = 0.65, *p* = 0.002). Humidity also emerged as a significant factor with a positive correlation (correlation coefficient = 0.48, *p* = 0.032), while phytosanitary risk and human intervention did not reach statistical significance.

Bootstrap methods were employed to assess confidence intervals and the stability of the results. For instance, the confidence interval for the mean risk level in the experimental group was 18.7−20.8%, indicating a high reliability of the obtained results. Correlation analysis revealed that temperature has a strong positive correlation with the risk level (correlation coefficient = 0.65, *p* = 0.002), emphasizing the importance of thermal conditions for the formation of a high-risk level. Humidity also proved to be a significant factor with a moderate positive correlation (correlation coefficient = 0.48, *p* = 0.032), highlighting the influence of humidity on the risk level in greenhouses.

The analysis demonstrated that the “Expert-Pro” model outperformed other AI models (such as the “Predictor” and “Precision+”) across all metrics, including accuracy, sensitivity, and specificity. Several key factors contributed to the superior performance of the “Expert-Pro” model.

“Expert-Pro” employs a recurrent neural network (RNN) architecture, which is particularly effective for time-series data and complex pattern recognition. This capability allowed the model to detect and predict pest risks based on subtle changes in environmental variables, such as temperature and humidity, over time.

Unlike other models, “Expert-Pro” utilizes adaptive learning algorithms that continuously adjust to changes in greenhouse conditions. This real-time adaptability enhances its ability to provide accurate risk assessments even as conditions fluctuate throughout the day and season.

The “Expert-Pro” model has a high sensitivity to initial indicators of pest presence, such as minor changes in plant physiological responses. This sensitivity enables early detection, giving greenhouse operators more time to intervene before infestations develop into larger issues.

“Expert-Pro” is optimized for multi-variable data processing, enabling it to effectively handle the diverse inputs from temperature, humidity, and pest activity sensors. This robustness supports the model’s high accuracy and reduces false positives, providing reliable alerts for pest management.

These advantages underscore why “Expert-Pro” is particularly suited for greenhouse pest monitoring, making it a practical tool for improving early detection and control efficiency in tulip cultivation.

## Discussion

Our study provides novel insights into the application of artificial intelligence (AI) for monitoring the risk level in tulip greenhouses. The results indicate that the use of artificial neural networks (ANNs) integrated into AI systems significantly enhances the prediction of the risk level. AI models, such as “Forecaster,” “Precision+,” and “Expert-Pro,” demonstrated high accuracy in predicting the risk level, making them effective tools for addressing agronomic challenges. Regression analysis identified internal temperature and humidity as key factors influencing the risk level in greenhouses. These findings underscore the importance of maintaining optimal thermal and humidity conditions for the successful cultivation of tulips.

The analysis of variance (ANOVA) revealed statistically significant differences among tulip varieties, emphasizing the need for an individualized approach to risk level control depending on the variety. The use of artificial intelligence (AI) and artificial neural networks (ANNs) also led to more effective pest control. The reduction in infestations of thrips, leafhoppers, and mites in the experimental group indicates that automated monitoring and management using AI contribute to combating pests at early stages.

The results of our study provide a foundation for the practical implementation of artificial intelligence (AI) in agriculture. Enhanced control over risk levels and pests will enable agricultural enterprises to reduce crop losses, utilize resources more efficiently, and enhance overall productivity. Based on the findings, a potential direction for future research could involve a more in-depth analysis of the impact of other factors, such as geographical conditions and seasonal variations, on the effectiveness of the control system. In conclusion, our study confirms the prospects of utilizing AI to optimize processes in agriculture, marking a significant step towards sustainable and efficient farming practices.

There are similar studies focusing on pest control and the application of artificial intelligence. For instance, a study by Böckmann et al. (2021) addresses the precise monitoring of pests in integrated pest management systems. The authors propose an effective method for differentiating whiteflies and their predators on sticky traps using a bag-of-visual-words algorithm (Böckmann et al. 2021). This work emphasizes the applicability of the developed system in real greenhouse conditions, aligning with the goals of our research on utilizing artificial intelligence for early pest detection in tulip greenhouses. Both studies aim to develop accessible and efficient tools for automated monitoring, enhancing the practical applicability of integrated pest management methods.

The study by Zhao et al. (2020) focuses on the automatic recognition of diseases in crops in field conditions using deep neural networks and the Internet of Things (IoT). The method achieves an accuracy of 97.5% in recognizing 77 diseases in field conditions, surpassing existing approaches.

The review by Fue et al. (2020) of robots for cotton harvesting in agriculture highlights opportunities and challenges for the development of robotic solutions in agriculture. Technologies focused on improving cotton harvesting in agriculture are discussed, emphasizing aspects such as mobility, sensing, route planning, and robotic grasping. Despite differences in focus, our work and the work of this group of authors highlight the prospects of applying modern technologies in agriculture. The study by Kusrini et al. (2020) proposes a machine-learning method for pest detection in mango fields using computer vision and deep learning. An accuracy of 76% was achieved on test data. In comparison with our research, it emphasizes the detection of pests in farmer conditions, utilizing data augmentation. The study by Zhang et al. (2021) provides an overview of the chemical ecology of entomopathogenic nematodes, identifying their responses to CO_2_ and substances emitted by plants and insects. In comparison with our research, which focuses on the application of artificial intelligence for monitoring and controlling pests in greenhouses, this study addresses the chemical mechanisms of target detection and recognition in entomopathogenic nematodes.

The study by Mahmud et al. (2023) provides a comprehensive overview of the application of sensor technologies, computer vision, artificial intelligence, and robotics in ornamental crop production. In the context of our research, which focuses on using artificial intelligence for monitoring and pest management in greenhouses, this study explores automation and sensor technologies in ornamental horticulture. The study by Abdullah et al. (2023) addresses the issue of pest and disease detection in agriculture using remote sensing, image analysis, and spectroscopy technologies. This is directly related to our research dedicated to the application of artificial intelligence for preventing diseases in greenhouses. The primary emphasis of this study is on the opportunities and challenges of monitoring pests and diseases using modern technologies. The study by Attri et al. (2023) covers the application of deep learning in agriculture, highlighting five main areas: crop yield prediction, plant stress detection, weed and pest detection, disease identification, and the development of smart agriculture. Key deep learning methods, such as Convolutional Neural Networks (CNN), Recurrent Neural Networks (RNN), AlexNet, and ResNet, are employed to enhance economic growth in agriculture. However, there is a recognized need for developing new deep-learning methods to improve model performance and reduce inference time for practical applications. The work underscores the importance of further research to fully unlock the potential of deep learning in smart agriculture and achieve sustainable agricultural development, aligning with the findings of our study.

The study by Abdulridha et al. (2020) applies hyperspectral imaging and machine learning to develop a methodology for detecting various stages of powdery mildew (PM) on pumpkins. Data were collected in laboratory and field conditions using an Unmanned Aerial Vehicle (UAV). To discriminate between healthy and diseased plants, as well as classify the degree of infection, a radial basis function (RBF) was employed. Significant wavelengths were identified for differentiation between healthy plants and various disease stages. The work emphasizes the effectiveness of certain spectral indices, such as the water index (WI) and the photochemical reflectance index (PRI), for accurate detection and classification of PM at different stages of development under laboratory conditions. Under field conditions using UAVs, efficient spectral indices for early and late disease development stages were identified. Classification results demonstrate high accuracy both in the laboratory and in the field, highlighting potential similarities with our study in the field of disease detection and classification using spectral data.

The study by Oberti and Schmilovitch (2021) emphasizes the importance of precise plant protection, with robotic systems playing a key role in the accurate monitoring and application of protective measures. This may be related to our research, where we utilized artificial intelligence, aiming for effective pest and disease management.

The study by Yang and Xu (2021) underscores the promise of deep learning in agriculture, particularly in handling large volumes of data. We observe parallels with our research, where artificial intelligence methods, including neural networks, were also employed for predicting the risk level, which could be a crucial element of intelligent farming practices.

## Conclusions

### Efficiency of artificial intelligence models

This study confirmed that the “Expert-Pro” model, a recurrent neural network (RNN), demonstrated the highest effectiveness in predicting pest risk levels in tulip greenhouses. Its performance across key metrics—accuracy, sensitivity, and specificity—surpassed that of the “Predictor” and “Accuracy+” models, showcasing its robustness in handling the complexity of pest dynamics influenced by environmental factors.

### Applicability in agriculture

The findings carry significant value for agricultural enterprises, particularly in greenhouse tulip cultivation. The “Expert-Pro” model’s high precision allows for more accurate risk assessments and timely interventions, reducing the reliance on chemical pesticides and minimizing crop losses. This AI-based approach aligns with sustainable agricultural practices, which are increasingly essential for eco-friendly farming and resource conservation.

### Control and experiment comparison

Comparative analysis between the control and experimental groups revealed a statistically significant reduction in pest risk levels within the experimental group. The success of AI-driven monitoring underscores its potential to enhance greenhouse pest management by reducing infestation rates and promoting plant health across various growth stages.

### Impact of temperature and humidity on risk levels

Temperature and humidity were identified as major factors influencing pest risk levels. High sensitivity of the AI model to fluctuations in these variables enabled more precise responses, crucial for adaptive pest management. In future applications, greenhouse operators could implement automated climate controls integrated with AI monitoring to maintain optimal conditions and proactively manage pest risks. This approach would contribute to more stable greenhouse environments and improve overall cultivation efficiency.

### Comparison of different tulip varieties

The study identified variations in risk susceptibility among tulip varieties, with certain types like “Albatros” and “Delta Storm” showing lower risk levels. This information is valuable for growers when selecting varieties for specific greenhouse environments, as it allows for strategic planning based on varietal resilience to pests.

### Practical applicability and recommendations

The research provides actionable insights for greenhouse operators and large agricultural enterprises seeking to implement AI in pest management. Practical recommendations include:

To fully leverage the predictive power of the AI model, greenhouses could integrate it with automated climate control systems. By synchronizing pest monitoring with temperature and humidity regulation, operators can better manage conditions that influence pest activity.

The data on varietal susceptibility can be applied to optimize pest management strategies by grouping lower-risk tulip varieties in high-priority areas.

Effective implementation requires training personnel in AI system operation and regular maintenance of sensors and cameras to ensure consistent data quality.

### Limitations and directions for future research

While this study highlights the efficacy of the “Expert-Pro” model, limitations include a restricted dataset and the focus on a specific AI model and crop type. Future research could benefit from:

Using a larger dataset across multiple seasons would improve the model’s generalizability and resilience against varied pest behaviors and environmental conditions.

Testing additional AI architectures, such as convolutional neural networks (CNNs) or hybrid models, may uncover models better suited to specific pest monitoring scenarios.

Extending the approach to other ornamental and edible crops in different greenhouse settings would validate its broader applicability.

Developing AI systems capable of initiating real-time adaptive responses, such as selective pesticide application or automated climate adjustments, would maximize resource efficiency and further reduce environmental impact.

This study underscores the transformative potential of AI in greenhouse pest management and offers a foundation for further advancements in sustainable and intelligent agricultural practices.

## Data Availability

The datasets generated during and/or analysed during the current study are available from the corresponding author on reasonable request.

## Abbreviations

AI: Artificial intelligence
ANFIS: Adaptive neurofuzzy inference system
ANNs: Artificial neural networks
AUC: ROC-area under the ROC curve
NPV: Negative predictive value
PM: Powdery mildew
PPV: Positive predictive value
PRI: Photochemical Reflectance Index
RBF: Radial basis function
UAV: Unmanned aerial vehicle
WI: Water Index
